# A Remarkable Genetic Diversity of Rotavirus A Circulating in Red Fox Population in Croatia

**DOI:** 10.3390/pathogens10040485

**Published:** 2021-04-16

**Authors:** Daniel Čolić, Nina Krešić, Željko Mihaljević, Tibor Andreanszky, Davor Balić, Marica Lolić, Dragan Brnić

**Affiliations:** 1 Virology Department, Croatian Veterinary Institute, Savska Cesta 143, 10000 Zagreb, Croatia; daniel.pmf5@gmail.com (D.Č.); lemo@veinst.hr (N.K.); miha@veinst.hr (Ž.M.); 2 Department of Biology, Faculty of Science, University of Zagreb, Rooseveltov trg 6, 10000 Zagreb, Croatia; 3 Veterinary Department, Croatian Veterinary Institute, Podmurvice 29, 51000 Rijeka, Croatia; andreanszky.vzr@veinst.hr; 4 Veterinary Department, Croatian Veterinary Institute, Josipa Kozarca 24, 32100 Vinkovci, Croatia; balic@veinst.hr (D.B.); lolic@veinst.hr (M.L.)

**Keywords:** *Rotavirus A*, red fox, genotype, molecular epidemiology, genetic diversity, phylogenetic analysis, interspecies transmission, Croatia

## Abstract

Rotaviruses (RV), especially *Rotavirus A* (RVA), are globally recognized as pathogens causing neonatal diarrhea, but they also affect intensive animal farming. However, the knowledge on their significance in wildlife is rather limited. The aim of the study was to unveil the prevalence, molecular epidemiology, and genetic diversity of RVA strains circulating in the red fox (*Vulpes vulpes*) population in Croatia. From 2018 to 2019, 370 fecal samples from fox carcasses hunted for rabies monitoring were collected. All samples were first tested using a VP2 real-time RT-PCR; in the subsequent course, positives were subjected to VP7 and VP4 genotyping. The results revealed an RVA prevalence of 14.9%, while the circulating RVA strains showed a remarkable genetic diversity in terms of 11 G and nine P genotypes, among which one G and three P were tentatively identified as novel. In total, eight genotype combinations were detected: G8P[14], G9P[3], G9P[23], G10P[11], G10P[3], G11P[13], G15P[21], and G?P[?]. The results suggest a complex background of previous interspecies transmission events, shedding new light on the potential influence of foxes in RVA epidemiology. Their role as potential reservoirs of broad range of RVA genotypes, usually considered typical solely of domestic animals and humans, cannot be dismissed.

## 1. Introduction

Rotaviruses (RV) are widespread pathogens of public health importance, causing an estimated 128,500 deaths each year, mostly in children in developing countries [1]. However, they also represent a substantial healthcare burden in developed countries [2]. The importance of RV infections in animals has mainly been recognized in cattle and pigs, since RVs are one of the main causative agents of neonatal diarrhea [3,4]. On the other hand, the knowledge on rotavirus infection in wildlife is rather limited [5].

Rotaviruses are a diverse group of enteropathogenic viruses of the *Rotavirus* genus belonging to the *Reoviridae* family. Among nine officially acknowledged species (*Rotavirus A–D*, *Rotavirus F–J*) [6], *Rotavirus A* (RVA) is by far the most significant from the human and animal health perspective [7]. The rotavirus dsRNA genome is enclosed within a triple-layered capsid and consists of 11 segments encoding 12 viral proteins, of which six are structural (VP1–VP4, VP6 and VP7) and six are nonstructural (NSP1–NSP6) [8]. As for the genotype nomenclature, the genome segments encoding the outer structural proteins VP7 and VP4 are used most, defining the G and P genotypes, respectively. However, nomenclature based on the genotype assignment to all 11 segments has been developed [9]. RVA is highly genetically heterogeneous, with 36 G and 51 P genotypes recognized by the Rotavirus Classification Working Group (RCWG) [10].

Due to the segmented nature of the genome, genetic reassortment is driving RVA diversification, with constant emergence of human–animal chimeric strains [3]. The examples of such events are numerous, involving primarily domestic animals [3]. However, a potential importance of RVA strains of wildlife origin in such an exchange of genetic material might have been underestimated, especially given that most of the emerging infectious diseases originate from wildlife [11]. Whether the marked genetic and antigenic diversity of RVA strains influences long-term vaccine effectiveness is a question that is still pending [7].

Among wild animals, red foxes (*Vulpes vulpes*) are especially interesting since they colonize urban and semi-urban areas and, therefore, may represent a disease spillover risk to human and animal health [12]. So far, the research on RVAs in red foxes comes down to one result obtained by negative-contrast electron microscopy [13] and the recent description of RVA as a causative agent of encephalitis [14]. The latter case is an interesting finding of an avian origin RVA strain affecting a mammalian host. To the best of our knowledge, the molecular epidemiology and genetic diversity of RVA strains circulating in foxes have not yet been studied. Therefore, the aim of our study was to report on prevalence, molecular epidemiology, and genetic diversity of RVA strains detected in the Croatian red fox population.

## 2. Results

### 2.1. The Results of VP2 Real-Time RT-PCR

Out of 370 tested samples, 55 were RVA-positive, resulting in a total RVA prevalence of 14.9% (95% CI, 11.4–18.9%). In study year 1 (2018), the RVA prevalence was 12.5% (24/192; 95% CI, 8.2–18%), while, in study year 2 (2019), it was 17.4% (31/178; 95% CI, 12.2–24%). The highest numbers of RVA-positive samples were detected in Osijek-Baranja (13/62), Vukovar-Srijem (8/62), Istria (7/29), and Sisak-Moslavina County (5/28). Overall, at least one positive sample was detected in 15 out of 16 studied counties in two sampling years (Table 1), with the sole exception of Lika-Senj County (0/6). Differences in prevalence between the two sampling years were not statistically significant (*p* = 0.184).

When we looked at the RVA prevalence in the group of 300 foxes of known age, the prevalence turned out to be slightly higher in animals under 12 months (19.3%, 17/88, 95% CI 10.2–29.1%) as compared to those older than 12 months (14.6%, 31/212, 95% CI 10.2–20.1%). However, the observed differences were not statistically significant (*p* = 0.313).

As for the Cq values of RVA strains detected in 2018 and 2019, 20 out of 24 and 27 out of 31 RVA-positive samples had a Cq value above 32, respectively. Overall, high Cq values (>32) were observed in 85.5% (47/55) of RVA-positive foxes. The results of amplification of exogenous internal positive control (IPC) RNA were positive in 98.1% of samples, indicating the absence of RT-PCR inhibitors. Seven samples that were IPC-negative in the first round of real-time RT-PCR were retested in 1:5 dilution and proved IPC-positive. These seven samples were negative for the presence of RVA genetic material.

### 2.2. The Results of VP7 and VP4 Genotyping and Phylogenetic Analysis

The G or P genotype was defined in 31 out of 55 RVA-positive foxes (15 in 2018 and 16 in 2019). The G genotype was successfully determined in 26 (14 in 2018 and 12 in 2019) and the P genotype was successfully determined in 14 RVA-positive foxes (seven in each study year). Most of the RVA strains detected during the course of the study were only partially genotyped (22/55 RVA-positive samples) or untypable (24/55 RVA-positive samples). Overall, the circulation of 11 G genotypes, specifically, G1, G2, G3, G5, G6, G8, G9, G10, G11, G15, and one tentatively novel G genotype, was detected. Moreover, nine P genotypes, specifically, P[3], P[11], P[13], P[14], P[21], P[23], and three tentatively novel P genotypes, were detected. In total, eight genotype combinations were detected in nine foxes, as follows: G8P[14] (*N* = 1), G9P[3] (*N* = 1), G9P[23] (*N* = 1), G10P[11] (*N* = 1), G10P[3] (*N* = 1), G11P[13] (*N* = 1), G15P[21] (*N* = 1), and G?P[?] (*N* = 2). The latter represents a tentatively novel G and P genotype combination, found in two foxes from Vukovar-Srijem County. It is important to acknowledge that some VP7 sequences (G1, G2, G3, G5, G6, and some representatives of the G8 and G9 genotypes) and all VP4 sequences do not meet the requirements for the correct genotype assignment proposed by the RCWG [9] and, therefore, may be considered as candidate G and P genotypes of the corresponding already established genotype.

#### 2.2.1. VP7 Genotypes and Phylogenetic Analysis

**Figure 1:**
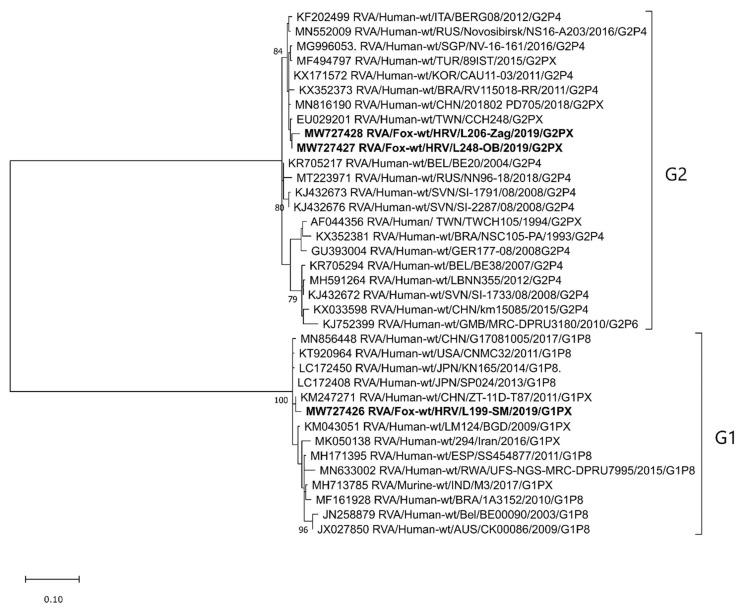
Phylogenetic relatedness of fox *Rotavirus A* (RVA) strains detected in Croatia with the reference RVA strains, determined on the basis of the VP7 genome segment (G1 and G2 genotypes). According to the alignment of shorter VP7 nucleotide sequences (~300 nt), the phylogenetic tree was generated using the MEGAX Software and the maximum-likelihood (ML) method by virtue of applying the T92 + G substitution model. The stability of the proposed branching order was assessed by bootstrapping (1000 replicates; indicated adjacent to the nodes when >70%). Fox RVA strains detected in Croatia are given in bold. The GenBank accession numbers of the selected RVA reference strains are designated within taxa. The scale bar represents the number of nucleotide substitutions per site. For the sake of simplicity in displaying the RVA strain nomenclature, P genotype numbers were omitted from the brackets.

The RVA strain of the G1 genotype was detected in two foxes from Vukovar-Srijem (2018) and Sisak-Moslavina (2019) Counties, for which we managed to obtain only shorter sequences, ~190 nt and ~300 nt, respectively. A partial sequence from the Sisak-Moslavina RVA G1 strain (RVA/Fox-wt/HRV/L199-SM/2019/G1P[X]) was included in the phylogenetic analysis and showed a close relatedness with a group of human RVA strains (Figure 1). At the nucleotide (nt) level, the identity with several human RVA strains was 98.3–99.0% and, at the amino acid (aa) level, it was 99.0–100%.

The G2 genotype was detected in 2019 in two RVA strains found in foxes from Osijek-Baranja and Zagreb Counties, for which we also managed to obtain only shorter sequences (~300 nt). As with G1, G2 turned out to be closely related to human RVA strains of the same genotype (Figure 1) (98.3–99.7% and 98.0–100% identity at the nt and aa levels, respectively).

The G3 genotype was detected in the RVA strain found in 2018 in a fox from Vukovar-Srijem County. In the same sampling year, the G5 genotype was confirmed in the RVA strain detected in a fox from Osijek-Baranja County, while the G6 genotype was found in the RVA strains detected in two foxes originating from Istria County. Due to the short sequence (~190 nt) generated by the American Group protocol [15], these genotype sequences could not be included in the phylogenetic analysis.

**Figure 2:**
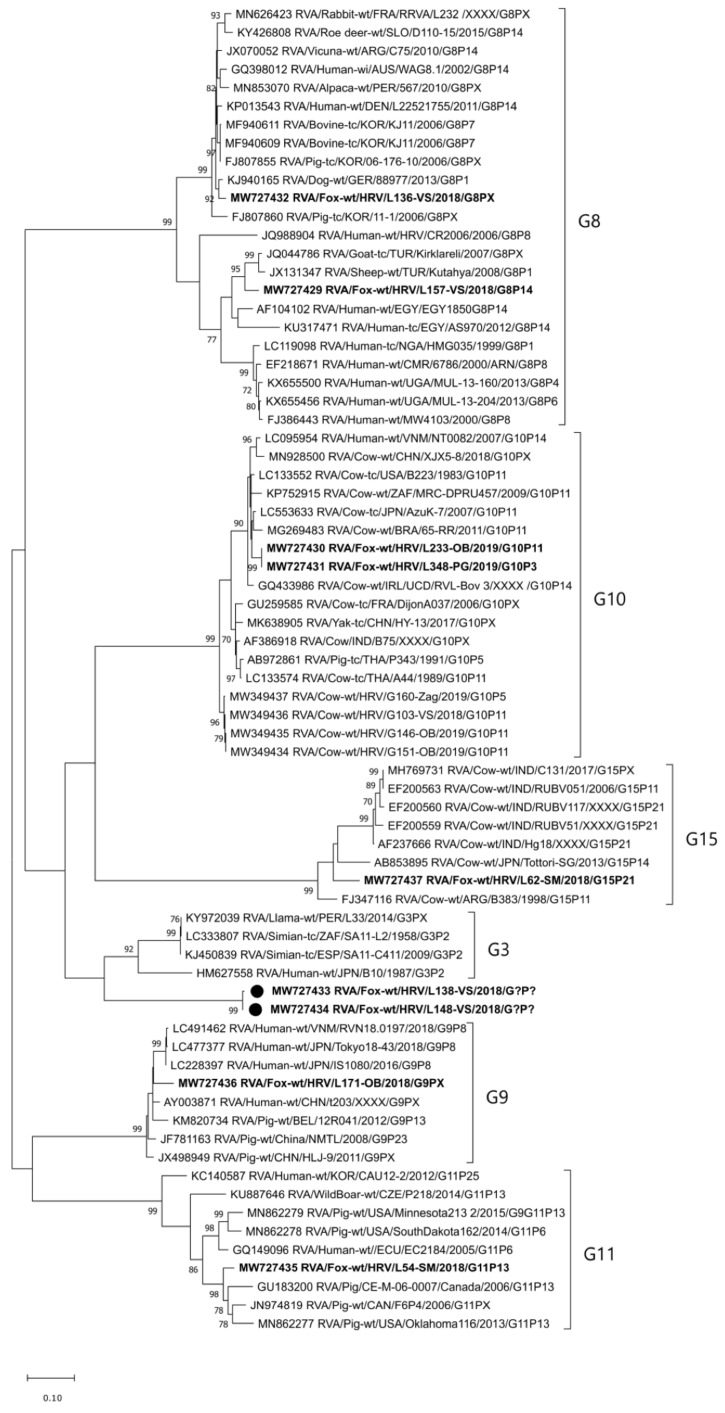
Phylogenetic relatedness of fox RVA strains detected in Croatia with the reference RVA strains, determined on the basis of the VP7 genome segment (G8, G9, G10, G11, and G15 genotypes and a tentatively novel one). According to the alignment of longer VP7 nucleotide sequences (~800 nt), the phylogenetic tree was generated using the MEGAX Software and the maximum-likelihood method by virtue of applying the T92 + G substitution model. The stability of the proposed branching order was assessed by bootstrapping (1000 replicates; indicated adjacent to the nodes when >70%). Fox RVA strains detected in Croatia are given in bold. The tentatively novel G genotype is represented by two RVA strains tagged with black dots. The GenBank accession numbers of the selected RVA reference strains are designated within taxa. The scale bar represents the number of nucleotide substitutions per site. For the sake of simplicity in displaying the RVA strain nomenclature, P genotype numbers were omitted from the brackets.

The G8 genotype was detected in the RVA strains detected in three foxes in 2018; two were from Vukovar-Srijem County and one was from Osijek-Baranja County. Two sequences included in the phylogenetic analysis had only 82.4% identity at the nt level (95.6% at the aa level), which is evident from their clustering into different lineages (Figure 2). The sequence of the RVA/Fox-wt/HRV/L157-VS/2018/G8P[14] strain is moderately similar to that of goat, sheep, bovine, and one human G8 RVA strain (91.2–93.3% at the nt and 97.2–97.6% at the aa level), while the RVA/Fox-wt/HRV/L136-VS/2018/G8P[X] strain shares high sequence identity with cow, dog, pig, alpaca, rabbit, vicuna, roe deer, and human strains (96.7–97.5% at the nt and 98.1–98.2% at the aa level) (Figure 2).

The G9 genotype was the most common RVA genotype detected in foxes in six counties (Table 1); two strains were detected in 2018 and seven were detected in 2019. Most sequences were partially of inadequate quality for a reliable phylogenetic analysis. The sequence included in the phylogenetic analysis (RVA/Fox-wt/HRV/L171-OB/2018/G9PX) was similar to pig and human RVA strains (93.1–93.7% and 94.9–95.7% identity at the nt and aa levels, respectively) (Figure 2).

The G10 genotype was detected in 2019 in the RVA strains found in two foxes originating from Osijek-Baranja and Primorje-Gorski kotar Counties. These two strains have a 100% sequence homology and are very similar to different bovine strains (96.9–97.1% at the nt and 98.9–99.3% at the aa level) (Figure 2). However, they form a separate lineage as compared to the autochthonous Croatian RVA strains of the same genotype, detected during 2018–2019 in cattle (MW349434-MW349437; 90.3–90.6% and 97.5–97.7% identity at the nt and aa levels, respectively) (Figure 2) and humans [16].

The G11 genotype was detected in the RVA strain found in a fox from Sisak-Moslavina County in 2018. Phylogenetic analysis showed its relatedness with RVA strains seen in domestic pigs (91.6–93.3% and 95.7–96.4% identity at the nt and aa levels, respectively) (Figure 2).

The G15 genotype was detected in the RVA strain found in a fox from Sisak-Moslavina County in 2018. This extremely rare genotype has only seven sequences in the NCBI GenBank, all of them of bovine origin. Phylogenetic analysis confirmed its relatedness with these sequences (Figure 2) in terms of nucleotide identity of 88.3% to 88.9% (sequence identity at the aa level was 93.2–93.5%). To the best of our knowledge, this is the first detection of the G15 RVA genotype in Europe.

The 11th G genotype detected in this study is a tentatively novel one, given that nucleotide identity was lower than 80% (79.2–79.4% with simian, simian-like human, and llama RVAs of the G3 genotype). The amino-acid sequence identity to these strains was 89.1–89.5%. This RVA genotype was detected in two foxes from Vukovar-Srijem County in 2018. These two sequences share 99.5% nucleotide identity (99.3% at the aa level).

#### 2.2.2. VP4 Genotypes and Phylogenetic Analysis

**Figure 3:**
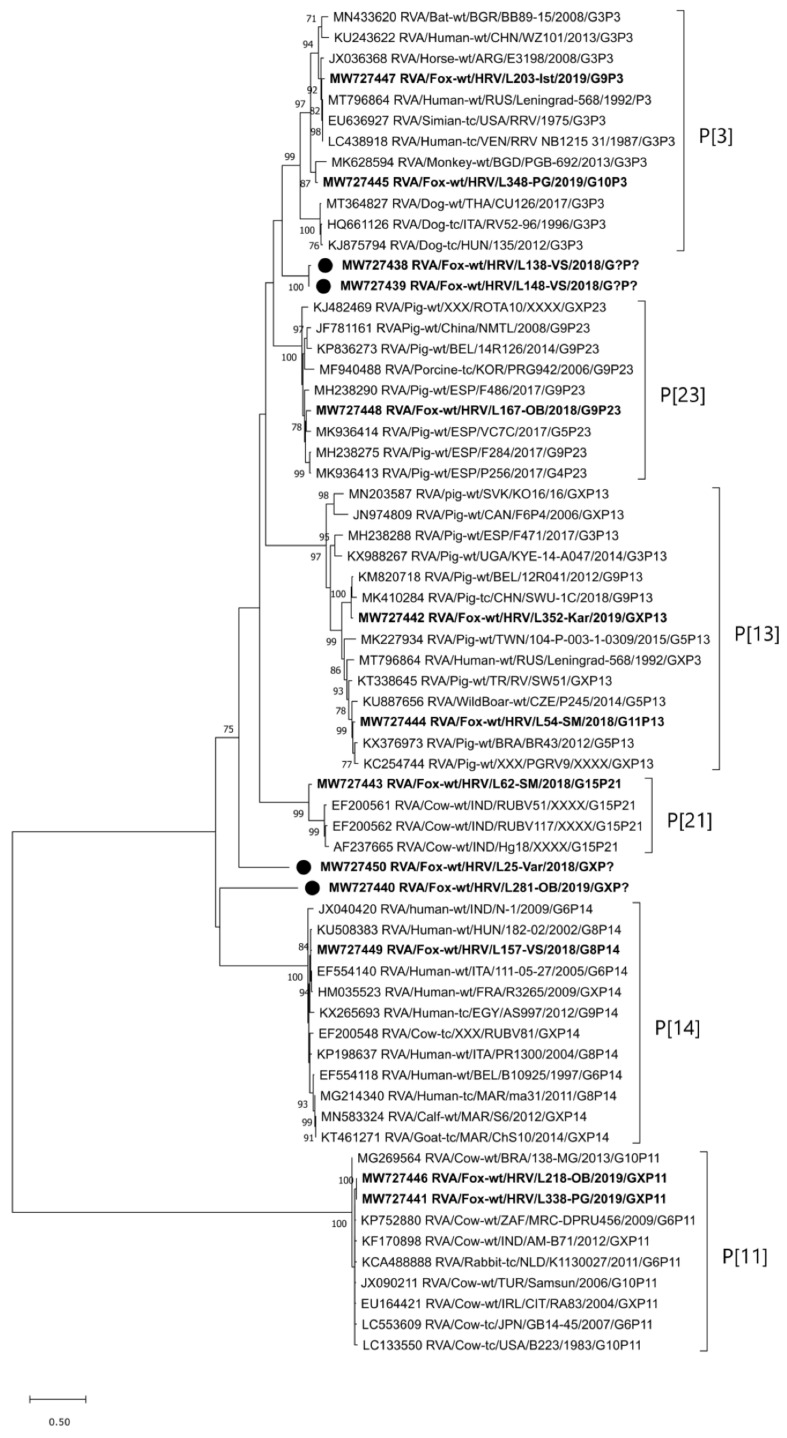
Phylogenetic relatedness of fox RVA strains detected in Croatia with the reference RVA strains, determined on the basis of the VP4 genome segment. According to the alignment of VP4 nucleotide sequences (~680 nt), the phylogenetic tree was generated using the MEGAX Software and the maximum-likelihood method by virtue of applying the TN93 + G substitution model. The stability of the proposed branching order was assessed by bootstrapping (1000 replicates; indicated adjacent to the nodes when >70%). Fox RVA strains detected in Croatia are given in bold. The RVA strains of tentatively novel P genotypes are tagged with black dots. The GenBank accession numbers of the selected RVA reference strains are designated within taxa. The scale bar represents the number of nucleotide substitutions per site. For the sake of simplicity in displaying the RVA strain nomenclature, P genotype numbers were omitted from the brackets.

The RVA strain of the P[3] genotype was detected in 2019 in two foxes from two neighboring counties, i.e., Istria and Primorje-Gorski kotar County. However, these two RVA strains are not closely related (only 86.2% and 94.2% identity at the nt and aa levels, respectively). The RVA/Fox-wt/HRV/L203-Ist/2019/G9P[3] strain shares high sequence identity to the group of human and simian RVAs (96.1–96.3% at the nt and 96.9–97.3% at the aa level), while the RVA/Fox-wt/HRV/L348-PG/2019/G10P[3] strain is most comparable to the MK628594 monkey RVA strain (91.2% at the nt and 91.0% at the aa level) (Figure 3). Interestingly, P[3] strains circulating in dogs branched in a different lineage (Figure 3). RVA strains of the genotype P[3] were in combination with the G9 (typical porcine and human genotype) and G10 genotypes (typical bovine genotype), indicating multiple previous interspecies transmission events.

The P[11] genotype was detected in the RVA strains found in three foxes in 2019; two of them were from Osijek-Baranja County and one was from Zagreb County. Two longer sequences were included in the phylogenetic analysis and were 100% homologous (Figure 3). The reference RVA strains, to which our fox strains are most closely phylogenetically related (97.6–97.8% and 98.7% identity at the nt and aa levels, respectively), are of bovine origin (Figure 3). The RVA strain of the genotype P[11] (RVA/Fox-wt/HRV/L233-OB/2019/G10[P11]), whose VP7 segment was successfully typed within this study frame, was in combination with the G10 genotype. Both genotypes involved in the G10P[11] combination are typical of cattle, indicating a possible common origin and interspecies transmission events.

The RVA strain of the P[13] genotype was detected in two foxes: one from Sisak-Moslavina County in 2018 and the other from Karlovac County in 2019. These two strains clustered in separate lineages (Figure 3) and they share only 85.3% and 83.9% identity at the nt and aa levels, respectively. However, both sequences showed close phylogenetic relatedness with and moderate to high sequence identity (93.8–97.2% and 91.9–97.3%) to, different porcine and wild boar RVA strains (Figure 3). The RVA/Fox-wt/HRV/L54-SM/2018/G11P[13] strain is a genotype combination typically found in pigs, suggesting a common origin and previous interspecies transmission events.

The P[14] genotype was identified in the RVA strain detected in a fox from Vukovar-Srijem County in 2018. This genotype was found in combination with the G8 genotype (strain RVA/Fox-wt/HRV/L157-VS/2018/G8P[14]), both considered to be of Artiodactyla origin. Nevertheless, this fox RVA strain showed the closest phylogenetic relatedness and the highest sequence identity with human RVA strains (98.5% at the nt and 98.5% at the aa level with the KU508383 strain detected in Hungary), as presented in Figure 3, indicating possible zoonotic potential.

The P[21] genotype of the RVA strain detected in a fox from Sisak-Moslavina County in 2018 is another extremely rare genotype identified within the frame of this study. It is also the first detection of this genotype outside India, since only three P[21] sequences are currently deposited in the NCBI GenBank. The genotype P[21] RVA strain (MW727443) detected in this fox is probably a sole member of a different lineage (Figure 3) since it shares only an 85.3–86.2% nucleotide level identity (90.2–92.0% at the aa level) to Indian strains detected in cattle. This fox RVA strain has the same G15P[21] genotype combination as the three RVA strains detected in India.

The P[23] genotype identified in the RVA strain found in a fox from Osijek-Baranja County in 2018 is typical of pig RVAs. This is also evident from the phylogenetic tree presented in Figure 3 and nucleotide sequence identity of 91.6–92.4% (95.3–95.8% at the aa level). The RVA/Fox-wt/HRV/L167-OB/2018/G9P[23] strain has the G9P[23] genotype combination, also typical of pigs, suggesting a common origin and previous interspecies transmission events.

Lastly, three tentatively novel P genotypes were detected in four RVA strains, given a nucleotide level identity of less than 80%. The tentatively novel P genotype identified in two RVA strains (RVA/Fox-wt/HRV/L138-VS/2018/G?P[?] and RVA/Fox-wt/HRV/L148-VS/2018/G?P[?]), detected in two foxes from Vukovar-Srijem County, has only a 70.2–76.4% nucleotide sequence identity (71.8–79.5% at the aa level) with some RVA strains of the P[3] and P[23] genotype. The second tentatively novel P genotype was identified in the RVA/Fox-wt/HRV/L25-Var/2018/GXP[?] strain detected in a fox from Varaždin County in 2018. It is distantly related to RVA strains of the P[3], P[21], P[23], and tentatively novel genotype detected in RVA strains L138-VS/L148-VS (60.5–67.8% and 60.4–72.6% identity at the nt and aa levels, respectively). The third tentatively novel P genotype was seen in the RVA/Fox-wt/HRV/L281-OB/2019/GXP[?] strain detected in a fox from Osijek-Baranja County in 2019. It is quite distantly related to other RVA strains included in the phylogenetic analysis (Figure 3). Sequence identity calculation suggests the highest sequence identity (59.5–61.9% at the nt and 55.7–62.0% at the aa level) with the tentatively novel P genotype strains referred to above (L138-VS, L148-VS, and L25-Var) and P[3], P[14], and P[23] genotypes. Nevertheless, clustering reliability will improve when more sequences are added to these newly proposed P genotype candidates.

## 3. Discussion

Rotavirus A is a widely distributed pathogen affecting a broad range of mammalian and avian hosts. Its genetic diversity is well researched in human strains [17], lagging in domestic animals [18], while it has only scarcely been investigated when it comes to wildlife [5]. This applies especially for foxes and their RVA strains, which have so far been described only twice: once in the 1980s using negative-contrast electron microscopy [13] and recently as a causative agent of encephalitis [14]. Therefore, the aim of this study was to provide new data on RVA prevalence, molecular epidemiology, and genetic diversity of RVA strains circulating in the Croatian red fox population.

The results of RVA detection using a VP2 real-time RT-PCR revealed that almost 15% of foxes are RVA-positive and widely distributed as such (circulating in 15 out of 16 counties under study). However, low Cq values (>32 in 85.5% of positive samples) indicate a predominantly low viral load in the tested fecal samples. This is not surprising given that all foxes included in the study were healthy (i.e., without any signs of gastrointestinal disease whatsoever) and mostly adult. The difference in RVA prevalence between the two age groups (19.3% in younger foxes, <12 months of age, and 14.6% in adult foxes, >12 months old) is not statistically significant. Nevertheless, the authors are aware that such comparisons call for a larger sample set, especially with regard to the younger fox group. Since this is the first study of RVA prevalence in red fox population, we are unable to put our results in a wider context and directly compare our results with those of other authors. However, studies on other wildlife species in a similar environment, which resorted to a similar methodological approach, indicated a considerably lower RVA prevalence in wild boars [19] and roe deer [20]. Moreover, the study on roe deer applied the same broad-range VP2 real-time RT-PCR designed for one of the most conserved regions of the RVA genome and tested on various human and animal samples [21].

With respect to the molecular epidemiology and genetic diversity of the RVA strains circulating in Croatian foxes, our results show an exceptional heterogeneity. This remarkable genetic diversity is evidenced by 11 G (G1, G2, G3, G5, G6, G8, G9, G10, G11, G15, and one tentatively novel) and nine P (P[3], P[11], P[13], P[14], P[21], P[23], and three tentatively novel) genotypes, together with eight genotype combinations (G8P[14], G9P[3], G9P[23], G10P[11], G10P[3], G11P[13], G15P[21], and G?P[?]) detected within this study frame. Some of these genotypes (G1, G2, G3, and G9) are among those most commonly found in humans [17]. Our results pertinent to the G1 and G2 genotypes (Figure 1) confirm a close relationship between fox and human RVA strains, indicating possible previous interspecies transmission events. Since these two genotypes are more common in humans [7], we cannot exclude the possibility of reverse zoonotic transmission from humans to foxes. A similar transmission route has already been hypothesized for RVA [22] and is well recognized for different microorganisms worldwide [23]. Nevertheless, we cannot exclude the possibility that foxes positive on G1 and G2 genotypes ingested food contaminated with RVA-positive human stool, and that the infection of fox intestinal cells never took place. The RVA strains of the G3, G5, and G6 genotype were not included in the phylogenetic analysis due to their short sequence. Nevertheless, we believe a trustworthy genotype determination was not compromised at any point [15], even though RCWG guidelines on genotype assignment [9] were not met. Some genotypes and genotype combinations detected in foxes indicate a common origin and possible interspecies transmission events involving RVA strains considered to be of porcine (G9P[23], G11P[13]) and bovine/Artiodactyla (G8P[14], G10P[11]) origin [7,18]. Some of these genotypes have already been detected in wildlife, for instance, the RVA G9, G11, P[13] and P[23] genotypes in wild boars [19,24]. Since foxes can surely come in contact with both domestic and wild animals and their excrements, the exact origin of interspecies transmission cannot be defined on the basis of sequence analysis only. Moreover, the lack of available sequences of wildlife origin in the NCBI GenBank further complicates reasoning. It is, however, important to emphasize that the majority of counties (12/16) from which the fox samples came belong to the Pannonian and North Croatia (Nomenclature of Territorial Units for Statistics; NUTS 2) regions, i.e., the country’s main livestock production regions. Nevertheless, small backyard holdings breeding cattle and pigs are present in all 16 counties (Table 1). These holdings, of usually lower biosecurity conditions, may serve as a contact point between livestock and wildlife species, which is the prerequisite for RVA interspecies transmission.

A potential background of multiple interspecies transmission events is evident in the RVA/Fox-wt/HRV/L203-Ist/2019/G9P[3] and RVA/Fox-wt/HRV/L348-PG/2019/G10P[3] strains. The G9 and G10 genotypes are considered to be typically porcine/human and bovine, respectively [7,18]. On the other hand, the P[3] genotype is predominant in dogs [7]. Interestingly, the P[3] genotype detected in foxes in this study branched separately from dog P[3] RVA strains in a lineage of multispecies origin (Figure 3). The genotype P[14] is sporadically detected in humans and is considered to originate from even-toed ungulates belonging to the mammalian Artiodactyla order [25]. A high resemblance of the fox P[14] RVA strain detected in our study to human strains (Figure 3) tempts one to speculate whether foxes may represent a reservoir of P[14] rotaviruses that affect humans. Another interesting study result is the circulation of G10 genotype in foxes, since this genotype is considered to be of bovine origin [18]. Our research on rotaviruses in cows and humans [16] indicated the circulation of the same genotype in the same geographical region (Osijek-Baranja County). However, the G10 RVA strains detected in foxes are somewhat distantly related to these strains and branch in a different lineage (Figure 2). Their high phylogenetic relatedness with various bovine RVA strains is evident (Figure 2), but the representatives of this G10 lineage in Croatian cattle have not yet been detected. This study revealed the presence of an extremely rare genotype combination G15P[21] (Figure 1 and Figure 3) in a fox from Sisak-Moslavina County. So far, only seven G15 and three P[21] genotype strains have been deposited into the NCBI GenBank. Since all strains of these RVA genotypes were detected in cows in India (G15 and P[21]) [26], Argentina (G15) [25], and Japan (G15) [27], we can assume bovine origin. Even though this strain is the first RVA G15 genotype detected in Europe and the first RVA genotype P[21] detected outside India, further research is needed to assess the impact of foxes as potential reservoirs of these RVA genotypes.

Perhaps the most interesting finding of this study is the detection of tentatively novel G (*N* = 1) (Figure 2) and P genotypes (*N* = 3) (Figure 3), as well as the detection of one tentatively novel genotype combination (strains RVA/Fox-wt/HRV/L138-VS/2018/G?P[?] and RVA/Fox-wt/HRV/L138-VS/2018/G?P[?]). Since this study is, to the best of our knowledge, the first molecular epidemiology study on RVA strains in foxes, it is not surprising that tentatively novel genotypes came up. They may also be species-specific; however, further research is warranted to corroborate this assumption. Since we obtained only partial VP7 and VP4 sequences of these genotypes, our work is currently oriented toward a complete CDS amplification required by the RCWG for the official recognition of novel genotypes [9]. This is of considerable importance for the strains of the tentatively novel G genotype, whose nucleotide level identity was found to be just below the defined 80% cutoff. Nevertheless, the application of whole-genome sequencing is critical for the elucidation of patterns of virus evolution [28].

Many of the detected RVA strains were only partially genotyped (22/55 RVA-positive samples) or untypable (24/55 RVA-positive samples), indicating difficulties in applying various typing approaches. These approaches mostly use standard sets of primers initially designed for genotyping human and domestic animal RVA strains [15,29,30,31,32,33]. The suggested low viral load witnessed in the majority of the tested samples poses as another obstacle to the goal.

The exact role of foxes in the epidemiology of certain RVA genotypes is currently unknown; however, a rising presence of foxes in urban and semi-urban settings may pose as a risk for disease spillover among humans and animals [12]. Our results indicate a possible role of foxes as RVA reservoirs of a broad range of genotypes usually considered typical of domestic animals and humans. Since the investigation into RVA strains in wildlife has so far been neglected by the scientific community, RVA genotypes typical of certain species, foxes included, are difficult to assess. Our results show a remarkable genetic diversity, which warrants further research to uncover the most prevalent RVA genotypes circulating among foxes. The complex background of interspecies transmissions highlighted by the present study emphasizes the need for continuous application of the One Health approach in rotavirus-devoted research.

## 4. Materials and Methods

### 4.1. Samples

Fecal samples from red foxes included in this study were collected from April to November 2018 (year 1) and from January to November 2019 (year 2) at the Croatian Veterinary Institute Pathology laboratories operating in Vinkovci (Vukovar-Srijem County), Zagreb (City of Zagreb County), and Rijeka (Primorje-Gorski kotar County). Feces was sampled directly from rectum of fox carcasses during annual sampling (brain, muscle, and teeth) for the purposes of monitoring the oral rabies vaccination (ORV) campaign organized by the Veterinary and Food Safety Directorate of the Croatian Ministry of Agriculture or for the purposes of rabies passive surveillance. Autopsy screening of all sampled foxes for the signs of diarrhea revealed no gastrointestinal diseases at all. A total of 370 samples (192 in 2018 and 178 in 2019) were collected from 16 Croatian counties, with the highest number of sampled foxes in the two easternmost counties (Osijek-Baranja County, *N* = 62 and Vukovar-Srijem County, *N* = 62). Counties not embraced by the study are mostly those located on the south coast. The age of fox carcasses collected for the ORV monitoring was estimated as described previously [34]. The age was successfully estimated in 300 sampled animals (81.1%). Accordingly, the foxes were classified as younger (*N* = 88) or older (*N* = 212) than 12 months. All fecal samples were stored at −20 °C until further use.

### 4.2. RNA Extraction and Real-Time RT-PCR

Prior to RNA extraction, a 20% *w/v* fecal suspension was prepared using Medium 199 (Sigma Aldrich, St. Louis, MO, USA). After a 3 min centrifugation at 14,000× *g*, the supernatant was used as a starting material for RNA extraction with MagMAX^TM^ CORE kit (ThermoFisher Scientific, Waltham, MA, USA) according to the manufacturer’s instructions (complex workflow). An exogenous internal positive control (IPC) RNA, Xeno™ RNA Control (ThermoFisher Scientific, Waltham, MA, USA), was added to each sample (2 µL) to monitor the presence of PCR inhibitors. The extraction protocol was automated by virtue of application of the KingFisher^TM^ Flex purification system (ThermoFisher Scientific, Waltham, MA, USA). The extracted nucleic acids were stored at −80 °C or processed immediately.

RVA RNA was detected using a real-time RT-PCR that amplifies a VP2 segment fragment of different RVA genotypes infecting humans and domestic animals [21]. Nevertheless, the VP2 protocol has successfully been applied to detect RVA RNA in wildlife, such as roe deer [20]. Prior to one-step real-time RT-PCR, the RVA dsRNA was denatured at 95 °C for 2 min together with primer mix (600 nM) and PCR-grade water. The reaction mixture combined a denaturation mix from the previous step, a VP2 probe (200 nM), the VetMAX™ Xeno™ Internal Positive Control (IPC)—VIC™ Assay (ThermoFisher Scientific, Waltham, MA, USA), and reagents contained by the VetMAX^TM^-Plus One-Step RT-PCR kit (ThermoFisher Scientific, Waltham, MA, USA). The reaction mixture setup and thermal cycling conditions were as recommended by the manufacturer. The runs were performed on a RotorGene-Q (Qiagen, Hilden, Germany). In cases of inhibition, the samples were diluted 1:5 and retested.

### 4.3. VP7 and VP4 Genotyping of RVA Strains

All samples that tested positive using VP2 real-time RT-PCR were subjected to genotyping in order to define G (VP7) and P genotypes (VP4). For that purpose, several different primer sets for VP7 and VP4 genotyping were applied (Table S1, Supplementary Materials), primarily due to the potentially high RVA genetic diversity coupled with possibly lower efficiency of previously designed primers.

VP7 genotyping made use of VP7-F and VP7-R primers alone or within the semi-nested protocol that combines VP7-F and VP7-RINT primers [29]. Another approach was the application of VP7 Beg9 and VP7 End9 primers [32] in the first round of RT-PCR, followed by nested PCR making use of VP7-up2 and VP7-down3 primers [30]. The last combination of primers used for VP7 genotyping was initially designed for low-RVA-load samples; N-VP7F1 and N-VP7R1 primers were used in the first RT-PCR round, while the primers N-VP7F2 and N-VP7R2 were used with the subsequent nested PCR [15].

For the sake of VP4 genotyping, the primers VP4_1-17F and VP4R_DEG [33] were initially applied, followed by VP4-HeadF and VP4-1094R2 [30] or, in some cases, VP4F and VP4R [29] or Rota-Seg4-s and Rota-Seg4-as [31]. As with VP7, the protocol designed for VP4 segment genotyping of low-RVA-load samples was used; primers N-VP4F1 and N-VP4R1 were involved in the first RT-PCR round, while primers N-VP4F2 and N-VP4R2 played their part in the second round of the nested PCR [15].

Reagents used with the RT-PCR were the SuperScript™ III One-Step RT-PCR System with Platinum™ Taq DNA Polymerase (ThermoFisher Scientific, Waltham, MA, USA), while those used with the nested/semi-nested PCR were GoTaq^®^ G2 Hot Start Colorless Master Mix (Promega, Madison, WI, USA). Primer concentrations and annealing temperatures used with each RT-PCR or nested/semi-nested PCR reaction were as recommended by the corresponding article. Other conditions, related to the reaction mixture setup and thermal cycling, were as recommended by the reagent’s manufacturer. Each reaction started with the initial dsRNA denaturation at 95 °C for 2 min, during which the extracted RNA was combined with the respective forward primer and PCR grade water. Hereafter, the remaining reagents were added into the reaction mixture run on the ABI 9700 GeneAmp thermal cycler (Applied Biosystems, Foster City, CA, USA).

### 4.4. Sequencing and Phylogenetic Analysis

RT-PCR or PCR products were purified prior to sequencing using an ExoSAP-IT™ PCR Product Cleanup Reagent (ThermoFisher Scientific, Waltham, MA, USA) or Monarch DNA Gel Extraction Kit (New England Biolabs, Ipswich, MA, USA). The latter was used as recommended by the manufacturer, while the former was used under modified conditions; 2 µL of ExoSAP-IT reagent was added per 20 µL of RT-PCR/PCR product and first incubated at 37 °C for 45 min and further at 80 °C for 15 min. Purified samples were sent to the Macrogen Europe (Amsterdam, the Netherlands) for direct Sanger sequencing in both directions. RVA genotypes were defined by a BLAST search (https://blast.ncbi.nlm.nih.gov/Blast.cgi) (accessed on 1 March 2021) based on the previously defined cutoffs [9].

Phylogenetic analysis included sequences of certain length and quality. As for the VP7 segment, we generated two phylogenetic trees. The phylogenetic tree displayed in Figure 1 was based on shorter sequences obtained within the semi-nested PCR protocol using VP7-F and VP7-RINT primers. The phylogenetic tree displayed in Figure 2 was based on longer sequences obtained within other VP7 RT-PCR (Table S1, Supplementary Materials). The only exception was the short sequence obtained with the application of VP7 nested RT-PCR, which was reliable enough for genotype assignment, but not informative enough for phylogenetic analysis [15]. The latter approach, which included shorter sequence genotype assignment, was also implemented for the VP4 segment [31]. Sequences obtained by other VP4 protocols described in Supplementary Table S1, with the exception of the VP4 nested RT-PCR [15], were suitable for a trustworthy phylogenetic analysis (Figure 3).

Multiple-sequence alignment (MUSCLE algorithm) and phylogenetic analysis were performed using the MEGAX Software [35] and the maximum-likelihood (ML) method by virtue of applying the T92 + G and TN93 + G substitution models (the lowest BIC score) for VP7 and VP4 sequence alignments, respectively. Clustering stability of the ML tree was evaluated using a bootstrap analysis with 1000 replicates. According to the previously aligned sequences, nucleotide and amino-acid pairwise identity matrices were calculated in R using the bio3d package [36,37]. VP7 and VP4 sequences were deposited into the NCBI GenBank under the accession numbers MW727426-MW727437 (292–872 nt) and MW727438-MW727450 (584–1032 nt), respectively.

### 4.5. Statistical Analysis

Statistical analyses were performed using the STATA 13.1, StataCorp. 2016 Stata Statistical Software: Release 13.1 College Station, TX: StataCorp LP. The results were tested for the normality of their distribution using the Shapiro–Wilk test and for the equality of variances using Levene’s test. In order to assess the statistical significance of differences in RVA prevalence between sampling seasons and age groups, the simple logistic regression and the nonparametric Spearman rank correlation test were used, respectively. Decisions on statistical relevance were made at the significance level of *p* ≤ 0.05.

## Figures and Tables

**Table 1:** The results of *Rotavirus A* (RVA) detection using a VP2 real-time RT-PCR and VP7 (G genotype) and VP4 (P genotype) genotyping, obtained across 16 Croatian counties.

County	The Number of RVA-Positive/Sampled Foxes			G Genotype	P Genotype
	2018	2019	Total		
Bjelovar-Bilogora	0/0	1/2	1/2	G9	
Brod-Posavina	0/1	2/7	2/8	G9	
Istria	4/18	3/11	7/29	G6, G9	P[3]
Karlovac	0/15	3/19	3/34	G9	P[13]
Krapina-Zagorje	1/15	1/2	2/17		
Lika-Senj	0/1	0/5	0/6		
Međimurje	0/1	1/6	1/7		
Osijek-Baranja	5/21	8/41	13/62	G2, G5, G8, G9, G10	P[11], P[23], P[?] *
Požega-Slavonia	0/4	1/2	1/6		
Primorje-Gorski kotar	1/14	2/19	3/33	G10	P[3]
Sisak-Moslavina	3/19	2/9	5/28	G1, G11, G15	P[13], P[21]
Varaždin	1/22	1/6	2/28		P[?] *
Virovitica-Podravina	1/4	0/5	1/9		
Vukovar-Srijem	7/34	1/28	8/62	G1, G3, G8, G9, G? *	P[14], P[?] *
Zagreb County	0/19	4/11	4/30	G2	P[11]
City of Zagreb	1/4	1/5	2/9		
Total	24/192	31/178	55/370		

* Question marks label tentatively novel G (*N* = 1) and P (*N* = 3) genotypes presented in Figures 2 and 3, respectively.

## Data Availability

The datasets used and/or analyzed within the frame of the study can be provided by the corresponding author upon reasonable request.

## Supplementary Materials

The following are available online at https://www.mdpi.com/article/10.3390/pathogens10040485/s1, Table S1: Primers used to genotype RVA strains detected in foxes.
