# Supplementary material for: A Remarkable Genetic Diversity of Rotavirus A Circulating in Red Fox Population in Croatia
Source: Pathogens. 2021 Apr 16;10(4):485. doi: 10.3390/pathogens10040485 (PMC8072941; doi:10.3390/pathogens10040485)
Supplement: Supplementary file 1 [file pathogens-10-00485-s001.zip › pathogens-1161147-supplementary.pdf]

Supplementary Table S1. Primers used to genotype RVA strains detected in foxes.

Segment/gene	Primer name	Annealing position (nt)	Primer sequence (5'→3')*	RT-PCR product size (bp)	Reference	No. of fox RVA sequences	Genotypes	No. of sequences included in the phylogenetic analysis
VP7	VP7-F<br>VP7-R	51-71<br>914-932	ATG TAT GGT ATT GAA TAT ACC AC<br>AAC TTG CCA CCA TTT TTT CC	881	[29]	10	G8 (N= 2),<br>G9 (N= 2),<br>G10 (N= 2),<br>G11 (N= 1),<br>G15 (N= 1),<br>G? (N= 2)[1]	9[a]
	VP7-RINT**	331-344	ANA YNG ANC CWG TYG GCC A	293	[29]	10	G1 (N= 1),<br>G2 (N= 2),<br>G9 (N= 7)	3 (G1, G2)[b]
	Beg9<br>End9<br>VP7-up2<br>VP7-down3	1-28<br>1036-1062<br>39-58<br>976-995	GGC TTT AAA AGA GAG AAT TTC CGT CTG G<br>GGT CAC ATC ATA CAA TTC TAA TCT AAG<br>GCT CCT TTT AAT GTA TGG TA<br>GAT CTY GAT CTY TTG GAC AT	1062<br><br>956	[32]<br><br>[30]	0	n/a	n/a
	N-VP7F1<br>N-VP7R1<br>N-VP7F2<br>N-VP7R2	37–58<br>354-370<br>49-71<br>220-242	TAG CTC CTT TTR ATG TAT GGT A<br>GTN GGC CAT CCT TTN GT<br>ATG TAT GGT ATT GAA TAT ACC AC<br>GTR TCC ATD GAT CCA GTN ATT GG	333<br><br>193	[15]	6	G1 (N= 1),<br>G3 (N= 1),<br>G5 (N= 1),<br>G6 (N= 2),<br>G8 (N= 1)	0
VP4	VP4_1-17F<br>VP4R_DEG	1-19<br>?***	GGC TAT AAA ATG GCT TCG C<br>TCY CTR TTR TAT TGC ATY TCY TTC C	~700	[33]	1	P[13]	1[c]
	VP4-HeadF<br>VP4-1094R2	1-27<br>1076-1101	GGC TAT AAA ATG GCT TCG CTC ATT TA<br>AAT GCT TGT GAR TCR TCC CAR TAA TC	1100	[30]	8	P[3] (N= 2),<br>P[11] (N= 2),<br>P[13] (N= 1),<br>P[21] (N= 1),<br>P[?] (N= 1)[1],<br>P[?] (N= 1)[2]	8[d]
	VP4-F<br>VP4-R	132-149<br>775-795	TAT GCT CCA GTN AAT TGG<br>ATT GCA TTT CTT TCC ATA ATG	663	[29]	4	P[14] (N= 1),<br>P[23] (N= 1),<br>P[?] (N= 1)[1],<br>P[?] (N= 1)[3]	4[e]
	Rota-Seg4-s<br>Rota-Seg4-as	766–788<br>1057–1078	TCT AAR ACA TCA TTN TGG AAR GA<br>GCT TGT GAA TCR TCC CAR TAA TC	312	[31]	1	P[11]	0
	N-VP4F1<br>N-VP4R1<br>N-VP4F2<br>N-VP4R2	1-20<br>236-257<br>10-32<br>203-224	GGC TAT AAA ATG GYT TCN YT<br>ARY ADC CAR TAA TCR NYD RGT G<br>ATG GYT TCN YTM ATT TAT AGA CA<br>GNT GGY TGA TAW GGA CCR TCK A	257<br><br>214	[15]	0	n/a	n/a

*N=A/C/G/T; R=A/G; D=A/G/T; Y=C/T; W=A/T; K=G/T; M=A/C
**VP7-RINT was applied in combination with VP7-F
***Annealing position was not provided by primer authors
[1]Two RVA strains (L138-VS and L148-VS) of the tentative novel G and P genotype
[2]RVA strain L281-OB of the tentative novel P genotype
[3]RVA strain L25-Var of the tentative novel P genotype
[a]GenBank accession numbers MW727429-MW727437
[b]GenBank accession numbers MW727426-MW727428
[c]GenBank accession number MW727444
[d]GenBank accession numbers MW727439-MW727443, MW727445-MW727447
[e]GenBank accession numbers MW727438, MW727448-MW727450
